# [Retracted] Long non‑coding RNA Igf2as controls hepatocellular carcinoma progression through the ERK/MAPK signaling pathway

**DOI:** 10.3892/ol.2024.14686

**Published:** 2024-09-18

**Authors:** Han Bao, Chun-Guang Guo, Peng-Cheng Qiu, Xin-Lei Zhang, Qi Dong, Yu-Kun Wang

Oncol Lett 14: 2831–2837, 2017; DOI: 10.3892/ol.2017.6492

Following the publication of this paper, it was drawn to the Editor's attention by a concerned reader that the flow cytometric data shown in Fig. 2C, the Hoechst 33342-stained images in Fig. 2D, the colony formation assay data shown in Fig. 3A, the cell invasion assay data in Fig. 3B and the cell proliferation assay data shown in Fig. 3C had already appeared in previously published articles written by different authors at different research institutes, or were featured in an article that was submitted for publication to a different journal at around the same time. Owing to the fact that the contentious data in the above article had already been published prior to its submission to *Oncology Letters*, the Editor has decided that this paper should be retracted from the Journal. The authors were asked for an explanation to account for these concerns, but the Editorial Office did not receive a reply. The Editor apologizes to the readership for any inconvenience caused.

